# Low-level laser therapy in temporomandibular joint disorders: a systematic review

**DOI:** 10.25122/jml-2020-0169

**Published:** 2021

**Authors:** Syed Ansar Ahmad, Shamimul Hasan, Shazina Saeed, Ateeba Khan, Munna Khan

**Affiliations:** 1. Department of Oral Surgery, Faculty of Dentistry, Jamia Millia Islamia, New Delhi, India; 2. Department of Oral Medicine and Radiology, Faculty of Dentistry, Jamia Millia Islamia, New Delhi, India; 3. Laboratory of Disease Dynamics and Molecular Epidemiology, Amity Institute of Public Health, Amity university, Noida, Uttar Pradesh, India; 4. Faculty of Dentistry, Jamia Millia Islamia, New Delhi, India; 5. Department of Electrical Engineering, Jamia Millia Islamia, New Delhi, India

**Keywords:** low-level laser therapy (LLLT), pain intensity, randomized controlled trials (RCTs), temporomandibular joint disorders (TMDs)

## Abstract

Temporomandibular joint disorders (TMDs) encompass a wide array of ailments affecting the temporomandibular joint (TMJ), muscles of mastication, and the allied structural framework. Myofascial pain, internal derangement of the joint, and degenerative joint diseases constitute the majority of TMDs. TMDs usually have a multifactorial etiology, and treatment modalities range from conservative therapies to surgical interventions. Low-level laser therapy (LLLT) has evolved as an efficient non-invasive therapeutic modality in TMDs. Previously conducted systematic reviews and meta-analyses have shown variable results regarding the efficiency of LLLT in TMJ disorder patients. Hence, this systematic review was carried out as an attempt to evaluate the efficacy of LLLT in the treatment of temporomandibular joint disorder patients.

## Introduction

TMJ disorders (TMDs) are categorized as degenerative musculoskeletal disorders causing structural and functional abnormalities [1]. Pain, diminished jaw functions and movements, midline deviation, malocclusion, joint noises, and locking constitutes the cardinal signs and symptoms of TMDs [2, 3]. The overall incidence of TMDs ranges from 21.5% to 50.5%, with a female gender predilection [4]. TMDs are categorized into three forms. Myofascial pain is the most typical form, followed by internal derangement of the joint and degenerative joint disease, respectively [5]. TMDs represent a primary cause of non-odontogenic pain in the orofacial region, with 40–75% of the individuals showing at least one TMD sign, such as TMJ noise, and 33% at least one symptom, TMJ or facial pain [6].Many TMDs may be self-limiting, with periodic remission and exacerbation of symptoms [7].

TMD therapies primarily aim to eliminate pain, joint clicking, restoring TMJ functions and entails dietary and behavioral amendments, pharmacotherapy, physical therapy, occlusal splint therapy, intra-articular injections, arthroscopy, arthrocentesis, Lasers, or open joint surgery [8]. Lasers have gained wide applications in dentistry owing to their therapeutic attributes, such as tissue healing and enhanced local microcirculation [9]. Low-level laser therapy (LLLT) refers to a light-based therapy that produces monochromatic and coherent light of a single wavelength [3].

LLLT may act via numerous mechanisms of action, including facilitating the release of endogenous opioids, augmenting tissue repair and cellular respiration, increasing vasodilatation and pain threshold, and decreasing inflammation [10]. LLLT exerts a photochemical effect, in contrast to the ablative or thermal effects related to medical laser procedures [11].

The current state of knowledge in LLLT as a therapeutic modality in TMDs is primarily based upon previously conducted prospective clinical trials, which have yielded debatable outcomes [12–16]. Few studies have demonstrated higher efficacy of LLLT over placebo [12, 15, 16], while others have shown similar efficiency of LLLT and placebo in the treatment of TMD [13, 14].

Many systematic reviews with or without meta-analyses have also demonstrated contentious results regarding the effectiveness of LLLT in TMDs [17–19]. A systematic review by Melis *et al.* demonstrated better efficacy of LLLT in eliminating TMJ pain as compared to the masticatory muscle diseases [20]. The meta-analyses by Gam *et al.* [21], Petrucci *et al.* [18], and McNeely *et al.* [22] could not establish the efficacy of LLLT therapy in TMJ pain. However, a meta-analysis conducted by Chang *et al.* suggested that LLLT has a reasonable analgesic effect on TMJ pain [19]. A meta-analysis by Chen *et al.* reported that LLLT might substantially enhance the functional outcomes with limited pain amelioration in TMD patients [23]. A systematic review with meta-analyses demonstrated that LLLT is not only effective in pain relief but also improves functional outcomes in TMD patients [4]. Few randomized controlled trials (RCTs) documenting the efficacy of LLLT in TMDs have been conducted since the last published systematic review [5, 11, 24–27].

However, to date, there is still no conclusive validation to substantiate or contradict LLLT for TMDs. Hence, this systematic review was conducted to substantiate and re-validate the efficacy of LLLT as a therapeutic modality in TMDs and review the evidence from previously published literature. The study results are also expected to serve as useful insight and guidelines for clinical practitioners treating patients with TMDs. This review will provide precise and obvious knowledge about the benefits and procedures of laser application, which have already been successfully established in TMD management.

Our objectives were to:

• Ascertain the efficacy of LLLT in pain diminution as the primary outcome and secondary outcome on TMJ functions, masticatory efficiency, psychological and emotional aspects;
• Compare LLLT with placebo and other interventions used in TMD management.

## Material and Methods

A systematic literature review was carried out to assess the efficiency of low-level laser therapy in patients with temporomandibular joint disorders.

### Research questions

The search for the systematic review was initiated by defining the keywords concerning the population, intervention, control, and outcomes (PICO) format: a) population – “temporomandibular joint disorders (TMDs)”; b) intervention/exposure – “low-level laser therapy (LLLT)”; c) control – “placebo or other interventions like occlusal splints, analgesics, transcutaneous electrical nerve stimulation (TENS) and botulinum toxins”; and d) outcome – “efficacy assessment”. The research question was designed for the above-mentioned keywords: a) “Is low-level laser therapy (LLLT) efficacious in patients with temporomandibular joint disorders”?

### Literature search and identification of studies

This search strategy followed the Cochrane guidelines for a systemic review. An extensive hand-searching and electronic searching were made between January 2000 to June 2020 using the combination of controlled vocabulary and free text terms in PubMed and Science direct search engines.

### Inclusion criteria

a) RCTs involving LLLT therapy in human subjects with TMDs; b) articles published in the English language between January 2000 to June 2020; c) at least a total of 10 study subjects (both LLLT and placebo categories).

### Exclusion criteria

a) Nonrandomized or crossover studies (studies other than RCTs); b) studies conducted on animal models; c) articles published in languages other than English and before January 2000; d) study subjects less than 10; e) studies that fail to provide information on the outcomes of interest and f) subjects with systemic disorders (i.e., rheumatoid arthritis and fibromyalgia) or non-TMD related pain (i.e., odontogenic pain, neuralgia, and psychological dysfunctions).

### Study selection

The titles and abstracts of the identified studies were thoroughly evaluated for potential eligibility. Studies that did not assess the efficacy of LLLT on TMDs were excluded. However, if the abstract of the study was unclear, the full texts of the study were then procured for evaluation. Manual cross-referencing of all the retrieved articles was carried out to identify any study missed previously.

### Outcome parameters

The primary outcome parameter was a diminution in the pain intensity in TMDs after LLLT therapy, expressed by the visual analog scale (VAS). The secondary outcome parameters were the effect on TMJ functions (expressed in terms of mouth opening, lateral and protrusive mandibular excursive movements, and TMJ noises), masticatory efficiency, pressure pain threshold (PPT), electromyographic (EMG) activity, quality of life (QoL), psychological and emotional aspects associated with TMDs.

### Data extraction

Data extraction was made based on the first author, year of publication, journal name, sample size, treatment design, type and wavelength of laser, dose and power of the used laser, study design, study outcome, and results. The included studies were reviewed by two other authors.

### Risk of bias assessment

The risk of publication bias was assessed by using the R-based Robvis software package introduced by the National Institute for Health Research (NIHR) (https://www.riskofbias.info/welcome/robvis-visualization-tool).

## Results

**Figure 1.**
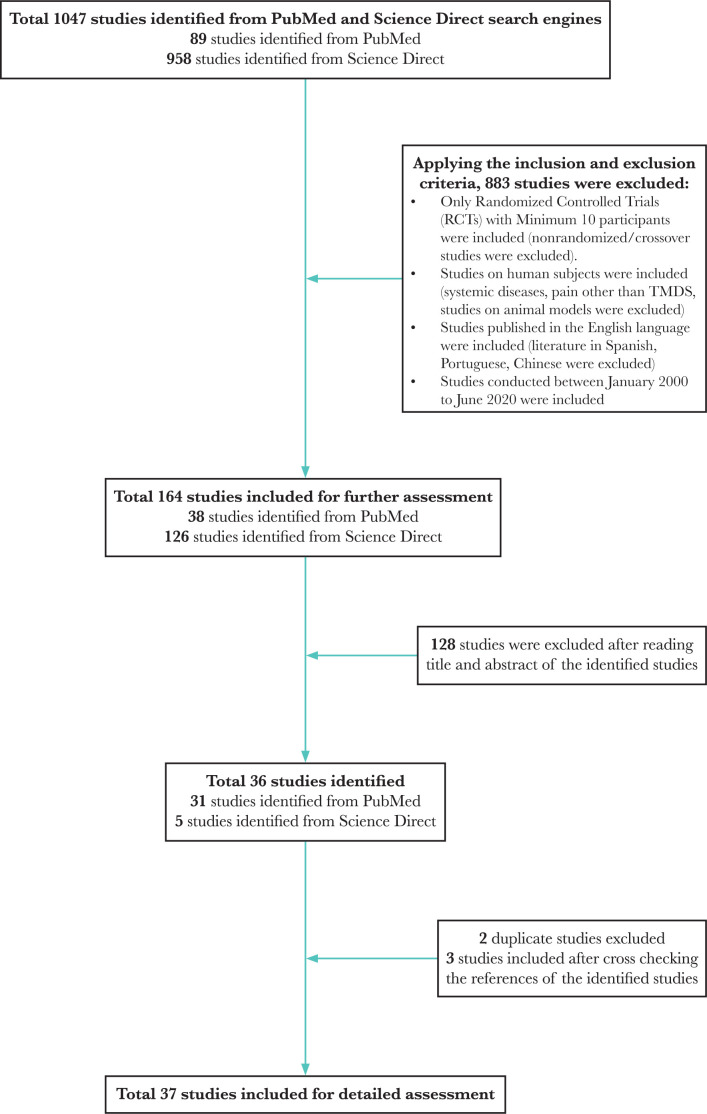
Selection of studies for the systematic review according to the PRISMA guidelines.

Thirty-seven articles were considered eligible for this systematic review. The selection cycle is in accordance with the Preferred Reporting Items for Systematic Reviews and Meta-Analyses (PRISMA) guidelines and is represented as a flowchart in Figure 1.

**Figure 2.**
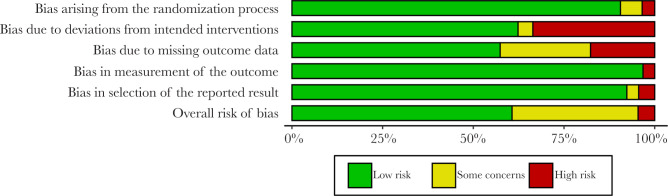
Robvis output for risk bias assessment.

**Figure 3.**
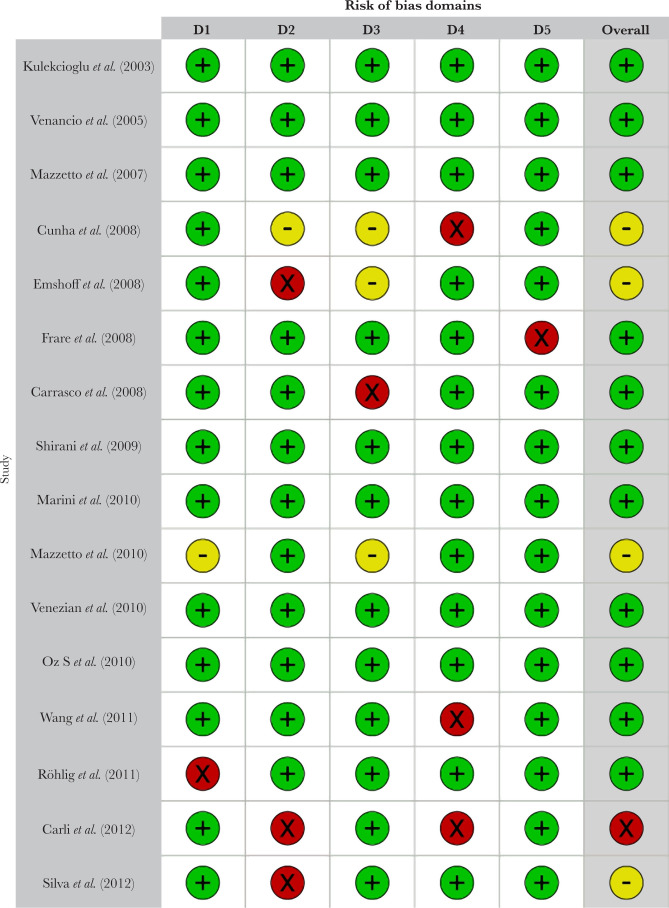

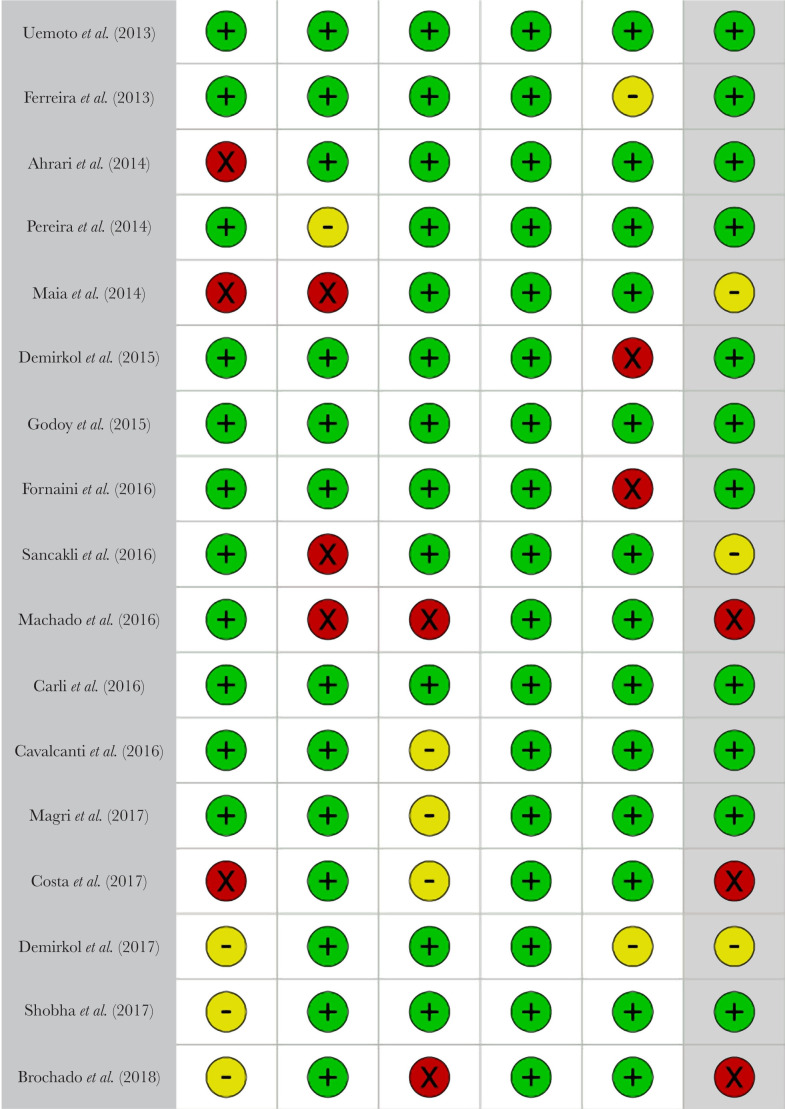

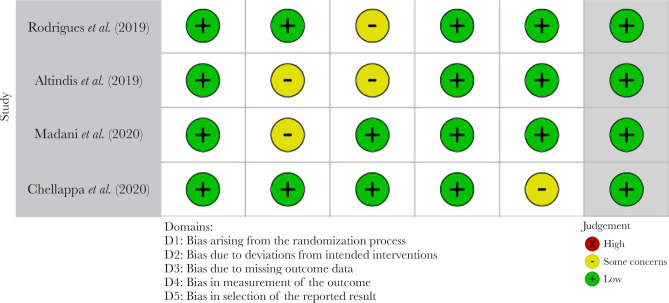
Weighted output for risk bias assessment.

Based on visual inspection of the figure generated by the Robvis software package, there is no potential publication bias in this study assessing the effectiveness of low-level laser treatment used in various RCTs for TMD patients (Figures 2 and 3). Out of 37 studies, 33 (89.18%) are high methodological studies, which have an overall low risk of bias or with some concerns, while only 4 studies have a high risk of bias. A detailed description of the eligible studies is given in Tables 1 and 2.

**Table 1.** Characteristics of the included studies.

Author	Sample size (n)	Age/gender	Treatment design	Type of laser, dose (j/cm^2^) and power (mw) of laser used	Outcome measures	Results
**Shobha *et al.* [5] (2017)**	n=40 Group 1 (Laser group n=20) Group 2 (placebo group n=20)	18–40 yrs Not mentioned	Laser (20) versus placebo (20)	Diode laser (gallium aluminum arsenide, 810 nm, 0.1 W, 6 J/cm^2^).	PI at function and at rest (VAS), MO and temporomandibular clicking	• ↓pain observed in both active LLLT and placebo groups • improvement in clicking
**Carli *et al.* [9] (2016)**	n=15 Group 1 (Laser group n=8) Group 2 (Botulinum toxin A n=7)	Mean age=28 yrs M: F=2:13	Laser (8) versus Botulinum toxin A (7)	GaAlAs 890 nm, 100 mW, 80 J/cm^2^	PI (VAS) and MO	Both Laser and Botulinum toxin A treatments were efficient in reducing pain, but laser therapy was much faster in pain diminution. (LLLT>Botulinum toxin A in pain resolution). However, both treatments showed no statistically significant improvement in MO.
**Ahrari *et al.* [10] (2014)**	n=20 Group 1 (laser group n=10) Group 2 (placebo group n=10)	Mean age 35.5 yrs, 20 Females	Laser (10) versus placebo (10)	GaAIAs 810 nm, 50 mW, 3.4 J/cm^2^	PI, mandibular movements	LLLT>placebo
**Chellappa *et al.* [11] (2020)**	n=60 Group 1 (LLLT group n=30) Group 2 (TENS group n=30)	Not mentioned	LLLT group (30) TENS group (30) n=60	672 nm diode laser 50 mW, 3 J/cm^2^	PI and range of mandibular motion	LLLT>TENS
**Ferreira *et al.* [12] (2013)**	n=40 Group 1 (laser group n=20) Group 2 (placebo group n=20)	20–40 yrs 40 females	Laser (20) versus placebo (20)	GaAIAs 780 nm, 112.5 J/cm^2^, 50 mW	PI	LLLT>placebo
**Emshoff *et al.* [13] (2008)**	n=52 Group 1 (Study group n=26) Group 2 (control-placebo n=26)	18–58 yrs M: F=10:42	Laser (26) versus placebo (26)	HeNe 632.8 nm, 1.5 J/cm^2^ and 30 mW	PI	LLLT=placebo
**Venancio *et al.* [14] (2005)**	n=30 Group 1 (Study group n=15) Group 2 (control-placebo n=15)	Not mentioned M: F=5:25	Laser (15) versus placebo (15)	GaAlAs 780 nm, 6.3 J/cm^2^ and 30 mW	PI, mandibular function, pain sensitivity	LLLT=placebo
**Marini *et al.* [15] (2010)**	n=99 Group 1 (Study/laser group n=39) Group 2 (ibuprofen n=30) Group 3 (control-placebo n=30)	Not mentioned	Laser (39) versus ibuprofen (30) versus placebo (30)	GaAIAs 910 nm, 400 mW	PI, mandibular function, morphologic structural analysis of TMJ	LLLT>placebo
**Wang *et al.* [16] (2011)**	n=42 Group 1 (Study group n=21) Group 2 (control-placebo n=21)	Not mentioned	Laser (21) versus placebo (21)	GaAIAs 650 nm/830 nm, 300 mW	PI, functional examination (MO, lateral and protrusive excursive movements)	LLLT > placebo
**Brochado *et al.* [24] (2018)**	n=51 Group 1 (photo biomodulation (PBM) group n=18) Group 2 (Manual therapy group n=16) Group 3 (Combined group n=17)	21–77 Yrs M: F=3:48	PBM group (18) Manual therapy group (16) Combined group (17)	PBM with 808 nm, 100 mW, 13.3 J/cm^2^	PI, mandibular movements, psychosocial aspects, and anxiety symptoms in TMD patients	All protocols tested were able to promote pain relief, improve mandibular function, and reduce the negative psychosocial aspects and levels of anxiety in TMD patients. However, the combination of PBM and MT did not promote an increase in the effectiveness of both therapies alone.
**Altindis *et al.* [25] (2019)**	n=20	18–45 yrs Not mentioned	Laser (10) stabilization splint (10)	N/A	PI, muscle sensitivity and the superficial skin temperature differences	Occlusal splint therapy and LLLT were effective in the treatment of MPS, and when thermographic data were considered, LLLT treatments could provide more advantageous results in these patients.
**Madani A. *et al.* [26] (2020)**	n=45 Group 1 (LLLT group n=15) Group 2 (LAT n=15 Group 3 Placebo group n=15)	Not mentioned	LLLT group (15) LAT group (15) Placebo group (15)	GaAlAs laser 810 nm, 200 mW, 21 J/cm^2^	The mandibular range of motion (Lateral excursive and protrusive movements) PI and Mouth opening	Both LLLT and LAT were effective in reducing pain and increasing excursive and protrusive mandibular motion in TMD patients. LAT could be suggested as a suitable alternative to LLLT, as it provided effective results while taking less chair time.
**Rodrigues *et al.* 27 (2019)**	N/A	Not mentioned	N/A	N/A	Physical and emotional symptoms in TMD patients	LLLT improved the physical and emotional symptoms of TMD, with results like splint therapy.
**Shirani *et al.* [28] (2009)**	n=16 Group 1 (Study group n=8) Group 2 (control-placebo n=8)	16-37 yrs M: F=4:12	Laser (the combination of two wavelengths, 8) versus placebo (8)	InGaAlP 660 nm and GaAs 890 nm, 6.2 J/cm^2^ and 1.0 J/cm^2^, 17.3 mW and 1.76 mW	PI	LLLT>placebo
**Demirkol *et al.* [29] (2017)**	n=41 Group 1 (Nd: YAG laser group n=15) Group 2 (diode Laser group n=16) Group 3 (placebo n=15)	Not mentioned	Nd: YAG laser (15) versus diode laser (16) versus placebo (15)	Nd: YAG laser (1064 nm), diode laser (810 nm), 250 mW, 8 J/cm^2^	The severity of the tinnitus (VAS)	LLLT>placebo
**Pereira *et al.* [30] (2014)**	n=19	21–55 yrs M: F=4:15	N/A	660 nm (red laser) and 795 nm (infrared) laser 8 J/cm^2^ in Muscles 4 J/cm^2^ in Joint	PI	Both lasers are effective in the treatment and remission of TMD symptoms
**Demirkol *et al.* [31] (2014)**	n=30 Group 1 (laser group n=10) Group 2 (occlusal splint group n=10) Group 3 (placebo n=10)	Not mentioned	Laser (10) versus occlusal splint (10) versus placebo (10)	Nd: YAG 1064 nm, 250 mW, 8 J/cm^2^	PI	LLLT>placebo
**Venezian *et al.* [32] (2010)**	n=48 Group 1 (Study group n=24) Group 2 (control-placebo n=24)	18–60 yrs M: F=5:43	Laser (24) versus placebo (24)	GaAIAs 780 nm, 25 J/cm^2^ or 60 J/cm^2^, 50 mW or 60 mW	PI and EMG Activity	LLLT>placebo (PI) LLLT=placebo (EMG Activity)
**Cunha *et al.* [33] (2008)**	n=40 Group 1 (Study group n=20) Group 2 (control-placebo n=20)	20–68 yrs Not mentioned	Laser (20) versus placebo (20)	GaAlAs 830 nm, 100 J/cm^2^ and 500 mW	PI and TMD status	LLLT=placebo
**Uemoto *et al.* [34] (2013)**	n=21 Group 1 (laser group n=7) Group 2 (needling group n=7) Group 3 (placebo n=7)	20–50 yrs 28 females	Laser (7) versus needling group (7) versus placebo (7)	Laser type N/A 795 nm, 4 J/cm^2^ or 8 J/cm^2^, 80 mW	PI, EMG activity, pain sensitivity, mandibular movements	LLLT>placebo (only 4 J/cm^2^)
**Oz S *et al.* [35] (2010)**	n=40 Group 1 (Study group n=20) Group 2 (control-occlusal splints n=20)	Mean age 32.8 yrs M: F=6:34	Laser (20) versus occlusal splints (20)	Laser type N/A 820 nm, 3 J/cm^2^ and 300 mW	PI, mandibular movements and pressure pain threshold	LLLT=occlusal splints
**Cavalcanti *et al.* [36] (2016)**	n=60 Group 1 (laser group n=20) Group 2 (PDP group n=20) Group 3 (placebo n=20)	20–50 Yrs 60 females	Laser (20) versus PDP (20) versus placebo (20	GaAlAs 780 nm, 30 mW, 35 J/cm^2^	Presence/absence of Pain	LLLT>placebo
**Carli *et al.* [37] (2012)**	n=32 Group 1 (Laser + piroxicam group n=11) Group 2 (laser + placebo piroxicam n=11) Group 3 (placebo laser + piroxicam n=10)	18–58 yrs M: F=3:29	Laser + piroxicam (11) versus laser + placebo piroxicam (11) versus placebo laser + piroxicam (10)	GaAlAs 830 nm, 100 J/cm^2^ and 100 mW	PI, functional examination (MO, lateral and protrusive excursive movements)	LLLT=placebo
**Fornaini *et al.* [38] (2015)**	n=24 Group 1 (laser group n=12) Group 2 (placebo group n=12)	17–64 Yrs M: F=5:19	Laser (10) versus placebo (10)	GaAs 904 nm, 15 mW, 6 J/cm^2^	PI	LLLT>placebo
**Mazzetto *et al.* [43] (2010)**	n=40 Group 1 (Study group n=20) Group 2 (control-placebo n=20)	Not mentioned	Laser (20) versus placebo (20)	GaAlAs 830 nm, 5 J/cm^2^ and 40 mW	PI, mandibular movements	LLLT>placebo
**Röhlig *et al.* [44] (2011)**	n=40 Group 1 (laser group n=20) Group 2 (control-placebo n=20)	Not mentioned	Laser (20) versus placebo (20)	GaAs 820 nm, 300 mW, 8J/cm^2^	PI, functional examination, pain sensitivity	LLLT>placebo
**Silva *et al.* [45] (2012)**	n=45 Group 1 (low energy level group n=15) Group 2 (high energy level group n=15) Group 3 (placebo n=15)	25–53 yrs M: F=15:30	Low energy laser (15) versus high energy laser (15) versus placebo (15)	GaAIAs 780 nm, 52 J/cm^2^ and 105 J/cm^2^, 70 mW	PI, mandibular movements	LLLT>placebo
**Sancakli *et al.* [46] (2016)**	n=30 Group 1 (laser group I n=10) Group 2 (laser group II group n=10) Group 3 (placebo n=10)	18–60 yrs M: F=9:21	Laser I (10) versus laser II (10) versus placebo (10)	GaAs 820 nm 30 mW, 3 J/cm^2^	PI, mandibular mobility, pain sensitivity	LLLT>placebo
**Costa *et al.* [47] (2017)**	n=60 Group 1 (photo biomodulation (PBM) group n=30)	18–76 yrs M: F=6:54	PBM group (30) versus placebo group (30)	infrared laser (830 nm) 100 mW, 100 J/cm^2^	Referred pain elicited by palpation and maximum mouth opening	PBMT (830 nm) reduces pain in algic points, but does not influence the extent of mouth opening in patients with myalgia
**Godoy *et al.* [48] (2015)**	N/A	14–23 yrs Not mentioned	Laser versus Placebo	Laser type N/A 780 nm, 50 mW, 33.5 J/cm^2^	PI, mandibular range of motion and occlusal contacts	No statistically significant differences were found regarding pain, mandibular range of motion, or the distribution of occlusal contacts after treatment with low-level laser therapy.
**Maia *et al.* [49] (2014)**	n=21 Group 1 (laser group n=11) Group 2 (placebo group n=10)	Mean age 27.7±1.44 yrs M: F=2:19	Laser (10) versus placebo (9)	GaAlAs 808 nm, 100 mW, 70 J/cm^2^	PI, masticatory performance, pain sensitivity	LLLT>placebo
**Magri *et al.* [50] (2017)**	n=91 Group 1 (laser group n=31) Group 2 (placebo group n=30) Group 3 (control n=30)	18–60 Yrs 91 females	Laser (31) versus placebo (30) versus control (30)	GaAlAs 780 nm, TMJ, 20 mW, muscle, 30 mW, 5 or 7.5 J/cm^2^	PI, pain sensitivity, the sensory and affective dimensions of pain	LLLT=placebo
**Carrasco *et al.* [51] (2008)**	n=14 Group 1 (Study group n=7) Group 2 (control-placebo n=7)	Not mentioned	Laser (7) versus placebo (7)	GaAlAs 780 nm 105 J/cm^2^ and 70 mW	PI and ME	LLLT>placebo (PI on palpation) LLLT=placebo (ME)
**Frare *et al.* [56] (2008)**	n=18 Group 1 (Study group n=10) Group 2 (control-placebo n=8)	18–45 yrs 18 females	Laser (10) versus placebo (8)	GaAs 904 nm 70 mW, 6 J/cm^2^	PI	LLLT>placebo
**Mazzetto *et al.* [57] (2007)**	n=48 Group 1 (Study group n=24) Group 2 (control-placebo n=24)	Not mentioned	Laser (24) versus placebo (24)	GaAIAs 780 nm 89.7 J/cm^2^ and 70 mW	PI	LLLT>placebo
**Kulekcioglu *et al.* [58] (2003)**	n=35 Group 1 (Study group n=20) Group 2 (control-placebo n=15)	20–59 yrs M: F=7:28	Laser (20) versus placebo (15)	GaAs 904 nm 3 J/cm^2^ and 17 mW	PI, mandibular function (Mouth opening: MO and LM), TMJ sounds	LLLT>placebo (MO, LM) LLLT=placebo (PI, TMJ sounds)
**Machado *et al.* [59] (2016)**	n=82	Not mentioned	GI: laser + Oral motor (OM) exercises (21) versus GII: pain relief strategies + OM exercises (22) versus GIII laser placebo + OM exercises (21) versus GIV: laser (18)	GaAlAs 780 nm, 60 mW, 60±1.0 J/cm^2^	PI, TMD severity, and orofacial myofunctional status	LLLT=placebo

F – Female; GaAlAs – Gallium-aluminum-arsenide laser; GaAS – Gallium-arsenide laser; HeNe – Helium-neon laser; LAT – Laser acupuncture therapy; LLLT – Low-level laser therapy; LM – Lateral movements; ND: YAG – Neodymium-doped yttrium aluminum garnet; M – Male; ME – masticatory efficiency; MPS – Myofascial pain syndrome; MO – mouth opening; MT – Manual therapy; N/A: Not Applicable; OM – Oral motor; PBM – Photobiomodulation; PI – Pain intensity; TENS – Transcutaneous electrical nerve stimulation; TMD – temporomandibular joint dysfunction; VAS – visual analog scale.

**Table 2.** Details of the eligible studies.

Author	Country of study	Journal	Treatment time/number of total sessions/number of sessions week	Site of laser application	Evaluation/follow-up
**Shobha *et al.* [5] (2017)**	India	Indian Journal of Dental research	60 s/8/2–3 per week	TMJ and muscles	Follow-up after 30 days
**Carli *et al.* [9] (2016)**	Brazil	Journal of Photochemistry and Photobiology, B: Biology	-/7/48 hours interval between each session	Muscles	N/A
**Ahrari *et al.* [10] (2014)**	Iran	Lasers in Medical Science	120 s/12/3	Muscles	Before intervention, after six applications, at the end of treatment, and 1 month after the last application
**Chellappa *et al.* [11] (2020)**	India	Indian Journal of Dental research	120 s/12/two sessions/week for 6 weeks	TMJ and muscles	N/A
**Ferreira *et al.* [12] (2013)**	Brazil	Lasers in Medical Science	90 s/12/1	TMJ and Muscles	Before intervention, monthly until intervention completed
**Emshoff *et al.* [13] (2008)**	Austria	Oral Surgery, Oral Medicine, Oral Pathology, Oral Radiology, and Endodontics	120 s/20/2–3	TMJ	Before treatment and 2, 4, and 8 weeks after the first laser therapy
**Venancio *et al.* [14] (2005)**	Brazil	Journal of Oral Rehabilitation	10 s/6/2	TMJ	Immediately before the first, third, and fifth treatment sessions, and at the follow-up appointments after 15, 30, and 60 days of the end of treatment
**Marini *et al.* [15] (2010)**	Italy	Clinical Journal of Pain	20 min/10/5	TMJ	PI at baseline, 2, 5, 10, and 15 days after treatment. Mandibular function at baseline, 15 days and 1 month after treatment. MRI at baseline and at the end of the treatment.
**Wang *et al.* [16] (2011)**	China	West China Journal	15 min/6/6	TMJ	Before treatment, immediately, 1 month and 2 months after treatment
**Brochado *et al.* [24] (2018)**	Brazil	Brazilian Oral Research	40 s (joint); 21min (muscle)/12/3 times a week for 4 consecutive weeks	TMJ and muscles	Follow-up after 4 and 8 weeks
**Altindis *et al.* [25] (2019)**	Brazil	Complementary Therapies in Medicine	N/A	Muscles	N/A
**Madani A *et al.* [26] (2020)**	Iran	Lasers in Medical Science	30 s/10/two times a week for 5 weeks	joint, muscles, and acupuncture points	Evaluated before treatment/after 5 sitting/10 sitting and 30 days after therapy
**Rodrigues *et al.* 27 (2019)**	Brazil	Complimentary Therapies in Medicine	N/A	TMJ and muscles	N/A
**Shirani *et al.* [28] (2009)**	Iran	Lasers in Medical Science	360 s/6/2	Muscles	Before and immediately after treatment, 1 week after treatment, and on the day of feeling complete pain relief
**Demirkol *et al.* [29] (2017)**	Turkey	Photomedicine and Laser Surgery	20 s or 9 s/10/5	External Auditory Meatus	Before treatment, immediately and 1 month after treatment
**Pereira *et al.* [30] (2014)**	Brazil	Cranio: The Journal of Craniomandibular and Sleep Practice	N/A	TMJ and Muscles	Reassessed at 24 hours and 30 days (short-term assessment), 90 days (medium-term), and 180 days (long-term)
**Demirkol *et al.* [31] (2014)**	Turkey	Lasers in Medical Science	20 s/10/5	Muscles	Before treatment, immediately and 3 weeks after treatment
**Venezian *et al.* [32] (2010)**		Cranio: The Journal of Craniomandibular and Sleep Practice	20 or 40 s/8/2	Muscles	PI: before treatment, immediately and 30 days after treatment EMG: before and immediately after treatment
**Cunha *et al.* [33] (2008)**	Brazil	International Dental Journal	20 s/4/1	TMJ and/or muscles	Before treatment and after the last treatment
**Uemoto *et al.* [34] (2013)**	Brazil	Journal of Oral Science	–/4/–	Muscles	Before treatment, after four sessions with intervals ranging between 48 and 72 h
**Oz S *et al.* [35] (2010)**	Turkey	Journal of Craniofacial Surgery	N/A	-/10/2 times per week	N/A
**Cavalcanti *et al.* [36] (2016)**	Brazil	Photomedicine and Laser Surgery	20 s/12/3	TMJ and Muscles	Before treatment, at each week till the fourth week after treatment
**Carli *et al.* [37] (2012)**	Brazil	Journal of Oral Rehabilitation	28 s/4/2	TMJ and Muscles	Before treatment, after the first, second, third, and fourth treatment sessions, and 30 days after last treatment.
**Fornaini *et al.* [38] (2015)**	Italy	Laser Therapy	15 min/14/7	TMJ	Before treatment, 1 and 2 weeks after treatment
**Mazzetto *et al.* [43] (2010)**	Brazil	Brazilian Dental Journal	10 s/8/2	TMJ	Before treatment, immediately, 7 and 30 days after applications
**Röhlig *et al.* [44] (2011)**	Turkey	Turkish Journal of Physical Medicine and Rehabilitation	10 s/10/3–4	Muscles	Before treatment and after the last applications
**Silva *et al.* [45] (2012)**	Brazil	Cranio: The Journal of Craniomandibular and Sleep Practice	30 s or 60 s/10/2	TMJ and/or Muscles	Before treatment, immediately after the first, fifth, tenth treatments, and 5 weeks after completing the applications
**Sancakli *et al.* [46] (2016)**	Turkey	BMC Oral Health	10 s/12/3	Muscles	Before treatment and after the completion of therapy
**Costa *et al.* [47] (2017)**	Brazil	Brazilian Oral Research	28 s/-/-	Muscles	Long-term evaluation (6 months)
**Godoy *et al.* [48] (2015)**	Brazil	Journal of Oral and Maxillofacial Surgery	20 s/-/-	Muscles	N/A
**Maia *et al.* [49] (2014)**	Brazil	Lasers in Medical Science	19 s/8/2	Muscles	MP and PPT, before treatment, at the end of treatment and 30 days after treatment VAS, at the same time as above; it was also measured weekly
**Magri *et al.* [50] (2017)**	Brazil	Lasers in Medical Science	10 s/8/2	TMJ and muscles	Before treatment, after each treatment and 30 days after last treatment
**Carrasco *et al.* [51] (2008)**	Brazil	Cranio: The Journal of Craniomandibular and Sleep Practice	60 s/8/2	TMJ	Before treatment, after the 8^th^ application, 30 days after the last application
**Frare *et al.* [56] (2008)**	Brazil	Revista Brasileira de Fisioterapia	16 s/8/2	TMJ and external auditory meatus	Before and immediately after all sessions of laser applications
**Mazzetto *et al.* [57] (2007)**	Brazil	Cranio: The Journal of Craniomandibular and Sleep Practice	10 s/8/2	TMJ (external auditory meatus)	Before treatment, after the 4^th^ and 8^th^ applications, and 30 days after the last application.
**Kulekcioglu *et al.* [58] (2003)**	Turkey	Scandinavian Journal of Rheumatology	180 s/15/–	TMJ and/or muscles	Before, after, and 1 month after treatment
**Machado *et al.* [59] (2016)**	Brazil	Lasers in Medical Science	45 min/12/1–0.5	TMJ and Muscles	Before treatment, immediately and 1 month after treatment

EMG – electromyography; MRI – magnetic resonance imaging; PI – Pain intensity; PPT – Pressure pain threshold; TMJ – temporomandibular joint; VAS – visual analog scale.

### Characteristics of the studies

Eighteen studies used the “Research Diagnostic Criteria” (RDC/TMD) for diagnosis of TMDs, followed by VAS in 6 conducted RCTs. 7 studies utilized a combination of these two diagnostic criteria. A wide variety of lasers were used in the included studies. Nineteen studies used a Gallium-aluminum-arsenide laser (GaAlAs). Gallium-arsenide laser (GaAs) was used in 5 studies. Neodymium-doped yttrium aluminum garnet (Nd: YAG), diode lasers, and red and infrared lasers were applied in 2 studies each, followed by Indium-gallium-aluminum-phosphide laser (InGaAlP) and Helium-neon laser (HeNe), which were used in one study each as shown in Table 1. A combination of two laser types was also used in 3 studies, namely that of Shirani *et al.* [28], Demirkol *et al.* [29], and Pereira *et al.* [30]. A single laser type at two different wavelengths (GaAlAs at 650 nm/830 nm) was used in an RCT by Wang *et al.* [16]. Single laser with two or three laser dosages was employed in 4 studies (Table 1).

The shortest and longest laser wavelengths used among the included studies were 632.8 nm [13] and 1064 nm [29, 31], respectively, except for Altindis *et al.* [25] and Rodriguez *et al.* [27], who did not mention the wavelength used in their lasers therapy. Laser dosage ranged between 1.5 J/cm2 to 112.5 J/cm2 for the majority of the studies. Laser power ranged between 1.76Mw [32] to 500mW [33]; 3 studies did not mention the power of the laser [25, 27, 30]. Temporomandibular joint and/or the affected muscles were the primary site of laser application in 18 of the conducted RCTs. Laser therapy was applied specifically at the TMJ in 9 RCTs. In 8 RCTs, the site of laser application was only in the muscles. In most of the conducted studies, laser application was made at pre-decided sites, irrespective of the fact that they were the points of maximum pain or not. However, in other RCTs, only the points of maximum pain intensity were irradiated (Table 2).

Most of the studies involved a comparison of LLLT and placebo groups. However, seven studies involved comparison of laser with other interventions, namely, botulinum toxin A [9], TENS therapy [11], ibuprofen [15], needling [34], occlusal splints [33, 35], physiotherapeutic and drug protocol (PDP) [36]. Two studies incorporated co-interventions equally to both LLLT and placebo groups. Piroxicam was incorporated with LLLT in one study [37], and in the other study, oral motor (OM) exercises were combined with LLLT [38].

Most of the included studies provided data on the primary outcome of laser therapy, like pain intensity. Eighteen studies focused on secondary outcomes like mouth opening (MO), followed by 13 studies on lateral excursive (LE) mandibular movements, 10 studies on protrusive excursive (PE) mandibular movements, 7 studies on PPT, and 2 studies each on EMG, joint noises, TMD related psychological and emotional aspects, masticatory efficiency (ME), respectively. One study each focused on subjective tinnitus and occlusal contacts distribution (Table 1).

Eighteen studies showed that LLLT was efficacious in diminishing TMD pain, whereas 12 studies showed that LLLT had similar efficacy as of placebo/controls/other intervention in TMD pain diminution. Four studies presented varied effects of LLLT on pain intensity, mandibular motion, EMG activity, and masticatory efficiency. Two studies revealed that LLLT improved the psychological and emotional aspects associated with TMDs, joint noises, masticatory efficiency, and EMG parameters, respectively. One study focused on subjective tinnitus, whereas another study suggested laser acupuncture (LAT) therapy as a suitable alternative to LLLT. The results demonstrate that LLLT appears to be efficient in diminishing TMD pain with variable effects on the outcome of secondary parameters (Table 1).

## Discussion

Orofacial pain/pain in the stomatognathic system region has a varied pathophysiological basis, and its diagnosis and therapy cover diverse aspects of medicine and dentistry. TMDs are one of the principal causes of orofacial pain. According to the International Association for the Study of Pain, TMDs are defined as an assembly of painful musculoskeletal disorders of the temporomandibular joints, masticatory muscles, and adjacent architecture [39].

The exact etiology of TMDs is still not completely elucidated; however, stress-induced repetitive jaw clenching and grinding accounts as the most important causative factor. Stress also plays a major role in sustaining and augmenting the TMD symptoms. TMDs pose significant diagnostic and therapeutic challenges owing to their multifactorial etiology, lack of investigative guidelines and strategies, and are widely considered as a physical, psychological, and functional disorder [40].

A vast majority of studies assessing TMD therapeutic protocols incorporate only pain scales (VAS) and MO analysis, thereby omitting other imperative characteristics like chronic pain, stress, anxiety, and depression. Dworkin and Le Resche later adopted the Research Diagnostic Criteria (RDC/TMD) in 1992 to overpower these discrepancies, and it also provided the academicians and practitioners with an effective and systematic method of examination, diagnosis, and classification of TMDs [24].

In our systematic review, 18 studies used RDC/TMD to diagnose TMDs. Six RCTs utilized VAS, whereas 7 studies utilized a combination of these two diagnostic criteria. TMDs generally have a gender predisposition, the disease predominantly affecting females (F:M = 2:1–8:1). Patients in the age group of 20 and 50 years are usually affected, an unusual age distribution for a degenerative disorder [1]. In our systematic review, most of the studies revealed a higher prevalence of TMDs among women compared to men with an age range between 20–55 years. Pain is the cardinal manifestation in TMDs. Pain in TMDs accounts for the most probable explanation of these patients seeking treatment. This also serves as a justification for most of the studies focused on assessing the efficacy of a wide array of therapeutic protocols with pain amelioration as the primary outcome [41]. Pain reduction also results in improved jaw motion, chewing, and masticatory efficiency [4]. The results in this systematic review were in coherence with the published literature, as most of the included studies in our review considered pain amelioration as the primary outcome of laser therapy.

Restriction or deflection in the range of mandibular movements (MO, LE and PE mandibular movements) and joint clicking are other frequent manifestations of TMDs. TMD patients also frequently report loss of masticatory efficacy. The masticatory patterns should be evaluated, and a definitive therapeutic protocol should be planned. Surface EMG, myofunctional procedure ratings, and assessment of masticatory efficiency are some of the employed objective approaches [42]. This systematic review also focused on improving the secondary outcomes like MO [5, 9, 10, 14–16, 26, 28, 30, 34, 37, 43–47], LE and PE mandibular movements [10, 14–16, 26, 28, 34, 37, 43–46, 48], PPT [14, 34, 35, 44, 46, 49, 50], EMG parameters [32, 34], joint noises [5, 28], TMD masticatory efficiency (ME) [49, 51], subjective tinnitus [29], and occlusal contacts distribution [48].

The importance of psychological factors (stress, anxiety, depression, and personality changes) has been thoroughly investigated in the etiopathogenesis of TMDs over the years. Published literature has demonstrated that the interrelation between stress, anxiety, depression, and distinct physical manifestations of TMDs is universally in sync with manifestations that are similar to those seen in other chronic musculoskeletal pain disorders [52]. Approximately 75% of TMD patients exhibit chronic features, with detrimental biopsychosocial outcomes like depression and somatization [12]. In our systematic review, two studies emphasized the role of LLLT in improving TMD-related psychological and emotional aspects [24, 27]. The World Association of Laser Therapy came to a consensus in 2004 on the design of clinical trials with LLLT in TMDs. According to the established protocol, the placebo group should compulsorily be a part of the study design [53]. Most of the included RCTs involved a comparison of LLLT and placebo groups. However, 7 RCTs involved a comparison of laser with other interventions or compared co-interventions equally to both LLLT and placebo groups (Table 1).

Therapeutic lasers are generally close to the electromagnetic radiation spectrum and vary from visible (red) to invisible (infrared) light. The most used wavelengths usually range between 600 and 1000 nm, permitting deeper penetration, relatively poor absorption, and easier transmission through the skin and mucous membranes [30].

In this systematic review, most of the studies used lasers with wavelengths within the electromagnetic radiation spectrum. The wavelengths ranged between 632.8 nm and 1064 nm. Only five studies used lasers with wavelengths in the red range (shorter than 780 nm). RCTs conducted by Altindis *et al.* [25] and Rodriguez *et al.* [27] did not mention the wavelength of the used lasers. Published literature has ascertained that combining lasers of two wavelengths have furnished positive outcomes. Lasers exert distinct effects in various biological tissues, explaining the variable results of laser therapy with different wavelengths [30]. In our systematic review, a combination of two laser types at different wavelengths was demonstrated by Shirani *et al.* [28], who used InGaAlP (660 nm) and GaAs (890 nm) lasers, Demirkol *et al.* [29], who used Nd: YAG (1064 nm) and diode laser (810 nm), and Pereira *et al.* [30], who used red laser (660 nm) and infrared laser (795 nm).

LLLT may show heterogeneity in the dose, power, and application time, with an irradiance of 5 mW/cm2 to 5 W/cm2, power range between 1 mW up to 10 W, with pulsed or continuous beams, and the application span of 30–60 s/point [54]. The measure of the laser effect is also determined by the laser dose. According to Bjordal *et al.* [55], the debate on the efficacy of LLLT in TMDs is primarily because of the variability in the laser dose. In our systematic review, laser dosage ranged between 1.5 J/cm2 to 112.5 J/cm2, except for 5 studies where data was not available (Table 1). Laser power ranged between 1.76 Mw [28] to 500 mW [35].

The included RCTs also showed a wide disparity in the frequency of laser application, the number of sessions/weeks, and the total number of laser sessions. The studies showed that the number of sessions per week ranged from 1–7. Most of the studies argued for 2 sessions per week [5, 11, 13, 14, 26, 28, 32, 35, 37, 43, 45, 49–51, 56, 57]. However, there was no mention of the number of sessions/weeks in a few studies [25–28, 30, 34, 47]. The total number of laser applications also showed great variance, ranging from 4 to 20 sessions. Eight studies argued for a total of 8 sessions [5, 32, 43, 49–51, 56, 57], followed by 12 sessions in by 7 studies [10–12, 24, 36, 38, 46], and 10 sessions in 6 studies [15, 26, 29, 31, 44, 45]. However, few studies provided no information on the total number of laser sessions [25, 27, 30, 35, 47, 48]. The time of laser application also varied widely in the included studies.

Kulekcioglu *et al.* recommended using LLLT as an alternative to other conventional treatment modalities in TMD of myogenic and arthrogenic origin [58]. However, Machado *et al.* suggested that combination therapy of LLLT and oral motor exercises are more efficient for the rehabilitation of TMD patients [59]. Studies using supplementary diagnostic aids – panoramic radiography (OPG), computed tomography (CT), and magnetic resonance imaging (MRI) – should be vigilantly evaluated, as the interpretations of these investigations may not always correspond with the signs and symptoms of TMDs [53].

Few studies in our review used auxiliary diagnostic methods for TMD diagnosis. TMJ imaging using CT and MRI was done in a study by Shirani *et al.* [28], and OPG was used in studies conducted by Shobha *et al.* [5], Venancio *et al.* [14], Venezian *et al.* [32], and Carrasco *et al.* [51]. Over the last few years, LLLT has evolved as an excellent intervention for TMDs, owing to its analgesic, anti-inflammatory, and regenerative effects with no documented unfavorable outcomes and exceptional patient compliance. However, there is still no conclusive validation to substantiate or contradict LLLT for TMDs. Here, we have attempted to upgrade the clinical validation for LLLT effects on TMDs [4]. The strengths of our systematic review were the large number of included RCTs, hence a larger sample size that was analyzed. Regarding the limitations of the review, published literature on the use of LLLT in TMDs has revealed contradictory outcomes, primarily due to the variation in laser dosage [19].

The primary limitation of this systematic review was that only two specific databases were searched (PubMed and Science Direct) due to limited access to databases. This study advocated performing another systematic review with meta-analyses by incorporating some more databases to strengthen the findings. The disparity in the treatment parameters (dosage, power, wavelength, number, and frequency of laser application) and within the patient sample are the other limiting factor of this review. Generally, LLLT yields better efficacy when used within the electromagnetic radiation spectrum, incorporating higher irradiation parameters (higher dose and power), a greater number of sessions, and frequency of applications [53].

## Conclusion

This systematic review aimed to re-validate the efficiency of LLLT in TMDs by thoroughly evaluating the previously conducted researches and further compare with placebo and other interventions. The study outcomes are expected to provide useful guidelines for practitioners treating patients with TMDs. The results demonstrate that LLLT appears to be efficient in diminishing TMD pain with variable effects on the outcome of secondary parameters. Also, LLLT provides advantages as the therapeutic regimen is non-invasive, reversible, with fewer adverse effects, and may also improve the psychological and emotional aspects associated with TMDs. Therefore, this systematic review highlights the role of LLLT as a promising therapeutic regimen for TMDs.

## Acknowledgments

### Conflict of interest

The authors declare that there is no conflict of interest.
